# Nanocellulose Wound Dressings with Integrated Protease Sensors for Detection of Wound Pathogens

**DOI:** 10.1021/acssensors.4c03428

**Published:** 2025-05-20

**Authors:** Olof Eskilson, Emanuel Wiman, Nina Reustle, Jakob Langwagen, Zeljana Sotra, Anna Svärd, Robert Selegård, Yağmur Baş, Linn Berglund, Kristiina Oksman, Torbjörn Bengtsson, Johan P. E. Junker, Hazem Khalaf, Daniel Aili

**Affiliations:** † Laboratory of Molecular Materials, Division of Biophysics and Bioengineering, Department of Physics, Chemistry and Biology (IFM), 4566 Linköping University, Linköping SE-58183, Sweden; ‡ Unit of Microbiology, Immunology and Reproductive Science, School of Medical Sciences, Faculty of Medicine and Health, 6233 Örebro University, Örebro 703 62, Sweden; § Centre for Disaster Medicine and Traumatology, Department of Biomedical and Clinical Sciences, Linköping University, Linköping 581 85, Sweden; ∥ Division of Materials Science, Department of Engineering Sciences and Mathematics, Luleå University of Technology, Luleå 971 87, Sweden; ⊥ Laboratory for Experimental Plastic Surgery, Department of Biomedical and Clinical Sciences, Linköping University, Linköping 581 85, Sweden

**Keywords:** protease, wound infection, nanocellulose, bacteria, gold nanoparticles

## Abstract

Wound infections result in delayed healing, morbidity, and increased risks of sepsis. Early detection of wound infections can facilitate treatment and reduce the need for the excessive use of antibiotics. Proteases are normally active during the healing process but are overexpressed during infection as part of the inflammatory response. Proteases are also produced by the bacteria infecting the wounds, making proteases a highly relevant biomarker for infection monitoring. Here, we show a fluorescence turn-on sensor for real-time monitoring of protease activity in advanced nanocellulose wound dressings for rapid detection of wound pathogens. Colloidal gold nanoparticles (AuNPs) were adsorbed on bacterial cellulose (BC) nanofibrils by using a carefully optimized self-assembly process. The AuNPs could either be homogeneously incorporated in BC dressings or 3D printed in wood-derived cellulose nanofiber (CNF) dressings using a BC-AuNP ink. The BC-adsorbed AuNPs were subsequently functionalized with fluorophore-labeled protease substrates. Cleavage of the substrates by proteases produced by the wound pathogens Staphylococcus aureus and Pseudomonas aeruginosa resulted in a significant increase in fluorescence that correlated with the growth phase of the bacteria. Wound dressing with integrated sensors for the detection of proteolytic activity can enable the sensitive and rapid detection of infections, allowing for optimization of treatment and reducing the risks of complications.

## Graphical abstract

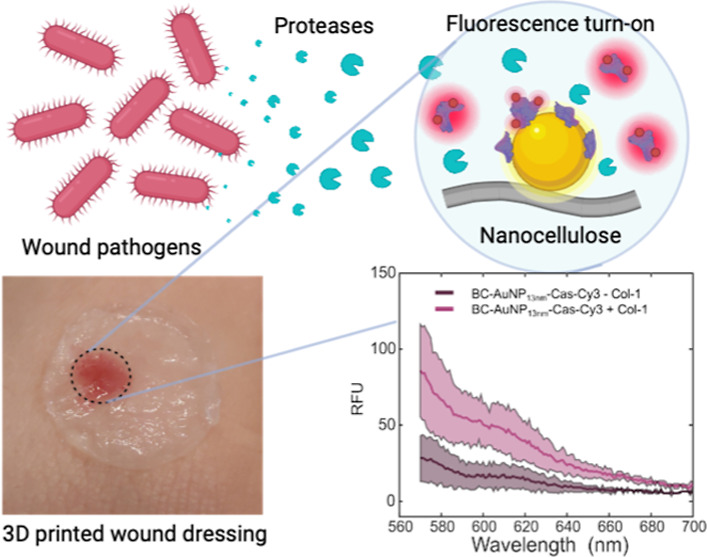

Wounds disrupt the normal barrier function of the skin which gives a plethora of pathogens the opportunity to colonize and spread throughout the underlying tissues.^1–3^ Wound infections can drastically impair the healing process and lead to wound chronification, tissue necrosis, and sepsis.^2,4–6^ Infections that prevents normal wound healing can have major negative effects on the quality of life of patients and give rise to persistent pain, distress, anxiety, and chronic morbidity.^4,7^ Nonhealing wounds impose a substantial burden on healthcare systems worldwide, accounting for 1–3% of total healthcare expenditures in developed nations. In the United States alone, the annual cost is estimated to range from $28.1 to $96.8 billion.^8,9^ Underlaying conditions, such as diabetes mellitus, obesity, and advanced age, make patients more susceptible to wound infections and wound chronification. The increasing prevalence of these conditions will increase the costs related to wound care in the coming decades.^8,9^ The development of advanced wound dressings based on alginate,^10–12^ chitosan,^13,14^ gelatin,^15^ fibroin,^16^ cellulose nanocrystals (CNC) and nanofibrils (CNF),^17,18^ or combinations of these,^19–21^ can provide moist conditions that can stimulate healing, but they do not typically prevent wound infections. Rapid detection and treatment of wound infections may lower the treatment costs, reduce the need for excessive use of antibiotics, and reduce patient suffering.^6^

Several sensing strategies have been developed for point-of-care detection of wound infections, using for instance nanoplasmonic sensors for infection biomarkers such as C-reactive protein and procalcitonin,^22^ as well as colorimetric,^23^ potentiometric^24^ and fluorometric pH sensors,^25^ and electrical^26^ and optical temperature sensors.^27^ Recently, hand-held fluorescence imaging devices have emerged as potential diagnostic tools that enable discrimination of infected and healthy tissues in a wound.^28^ Unfortunately, these techniques require removal of the dressing, which can be painful, disturb the wound healing process, and make the wound more susceptible to new pathogens. An alternative and less invasive strategy to monitor wound status is to integrate the sensors directly in the wound dressing.^29,30^ Wearable sensors integrated in wound dressings have been designed for monitoring of pH,^14,15,31–34^ uric acid,^35^ temperature,^36^ and oxygen levels,^17^ which can provide certain information about the healing process and the state of infection. However, detection of protein-based infection biomarkers, such as proteases, remains challenging using current dressing-integrated sensor strategies.

Proteases, primarily matrix metalloproteinases (MMPs), have a critical role in the wound healing processes, controlling the balance between formation of new tissue and tissue degradation by, e.g., remodeling of the extracellular matrix (ECM) and activation of fibroblasts and growth factors.^37–39^ Excessive proteolytic activity may, however, cause adverse effects and disrupt the balance between tissue remodeling and degradation, prolonging the inflammatory phase and potentially leading to wound chronification.^40,41^ Bacteria both secrete their own proteases and activate endogenous MMPs, resulting in aggravated tissue degradation.^42–44^ Since infection results in a significant upregulation of proteolytic activity in the wound, it is consequently a highly relevant biomarker for early detection of wound infections.^40,45^ Numerous strategies have been developed to quantify protease activity in wound fluids, using lateral flow devices^46^ and microfluids devices coated with multilayered fluorogenic nanofilms.^47^ These technologies are, however, not possible to integrate in the dressings and require dressing removal and sampling prior to analysis.

Here, we present a novel sensor for protease activity monitoring that is integrated into advanced hydrogel wound dressings composed of bacterial nanocellulose (bacterial cellulose (BC), Epiprotect) and wood-derived cellulose nanofibrils (CNFs) (Figure 1), enabling rapid detection of two of the most prevalent bacterial pathogens in chronic wound infections, Staphylococcus aureus and Pseudomonas aeruginosa. S. aureus and P. aeruginosa show distinct virulence mechanisms, biofilm formation capabilities, and antimicrobial resistance profiles that contribute to delayed wound healing and increased morbidity. To enable early detection of wound infections, we functionalized nanocellulose wound dressings with nanoplasmonic gold nanoparticles (AuNPs) that were subsequently modified with fluorescently labeled protease substrates, creating a “turn-on” fluorescence sensor for real-time detection of protease activity (Figure 1). Prior to proteolytic cleavage, the short separation between the fluorophore and the AuNP surface resulted in efficient fluorescence quenching. Proteases secreted by wound pathogens triggered a degradation of the immobilized substrates, which resulted in a release of the fluorophores, turning the dressings fluorescent. The nanocellulose dressings show excellent conformability to the wound surface and provide a moist wound microenvironment that promotes healing.^48,49^ The nanofibrillar structure of the dressings allows for efficient water vapor transmission and gas exchange while acting as a physical barrier that can prevent bacterial penetration.^50,51^ In contrast to conventional dressings that typically must be changed 1–2 times per weeks, the BC-based dressings can stay on the wound for several weeks, which circumvents painful and costly dressing changes.^52^ However, the dressings are not antimicrobial, and infections can result in complications and require dressing removal. The proposed wound dressing-integrated sensor technology for monitoring of protease activity offers a noninvasive approach to detect early signs of wound infection, without the need for dressing removal. Leveraging portable fluorescence readers already available in clinical practice, this technology has the potential to improve patient outcomes, reduce unnecessary antibiotic use, and significantly enhance the quality of life for individuals suffering from hard-to-heal wounds.

**Figure 1:**
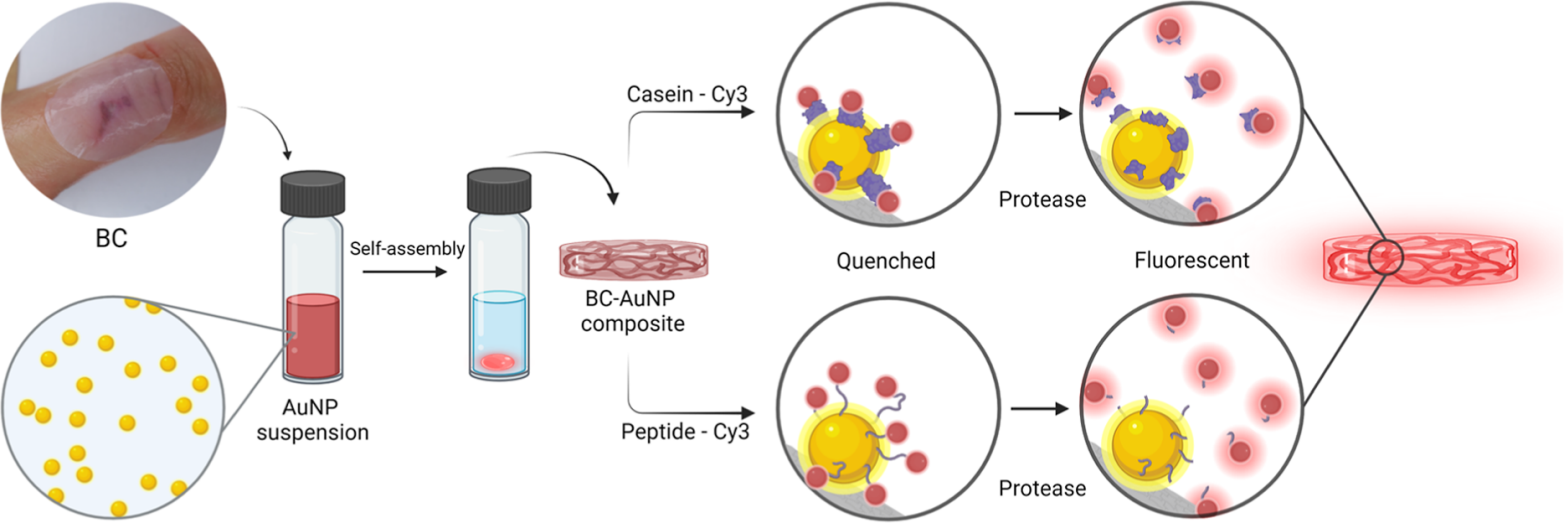
Photograph of a BC wound dressing and a schematic representation of the functionalization strategy for generating protease-responsive BC-AuNP nanocomposite dressings.

## Results and Discussion

### Fabrication of Nanocellulose-AuNP Composite Wound Dressings

AuNPs have been widely used as transducer elements in wearable sensors for healthcare applications because of their unique optical properties, chemical stability, and low toxicity.^53^ Here, we used citrate-stabilized colloidal AuNPs of two different sizes (ø 13 nm: AuNP_13 nm_ and ø 50 nm: AuNP_50 nm_) for functionalization of nanocellulose wound dressings to generate a dressing-integrated platform for protease activity monitoring. We have previously demonstrated that AuNPs bind strongly to BC nanofibrils at sufficiently high ionic strength as a result of the compression of the electric double layer that otherwise stabilizes the colloids.^54^ When reducing the repulsive electrostatic interactions, the AuNPs and the BC can come in close contact, allowing for an AuNP adsorption process that occurs on experimental time scales through an activated process that is primarily driven by short-range van der Waals attraction. Suspending the AuNPs in 10 mM citrate buffer pH 6 resulted in an adequate balance between colloidal stability and adsorption rate to the BC, resulting in wound dressings with a bright red color and a homogeneous distribution of AuNPs on the nanofibrils (Figure 2a). Ultraviolet–visible (UV–vis) spectra of BC functionalized with AuNPs showed distinct and well-defined localized surface plasmon resonance (LSPR) band with maxima at 520 and 531 nm for 13 and 50 nm AuNPs, respectively (Figure 2b). Aggregation of AuNPs in suspension or on surfaces results in a red-shift and broadening of the LSPR band due to the electromagnetic coupling between adjacent particles and the resulting changes in the dielectric environment.^55,56^ Here, the position and appearance of the LSPR band showed that the separation between the immobilized AuNPs was large enough to prevent optical coupling, which was further confirmed by scanning electron microscopy (SEM) (Figure 2c,d). The SEM showed that larger quantities of the 13 nm AuNPs were absorbed compared to the 50 nm AuNPs, which was further confirmed by the UV–vis spectra of the BC-AuNP dressings. The extinction cross section of the 50 nm AuNPs is more than 50 times larger than that for the 13 nm AuNPs while the LSPR intensity was 2.5 times higher for BC-AuNP_13 nm_ (Figure 2b), indicating that the amount of AuNPs is about 100 times higher in BC-AuNP_13 nm_ compared to in BC-AuNP_50 nm_.^57^ The difference in the amount of adsorbed AuNPs is likely a result of the lower concentration and slower diffusion rate of the larger AuNPs. Irrespectively of size, the AuNPs could be homogeneously integrated in the entire BC dressings, which can facilitate both sensing with high spatial resolution and sensitivity. However, due to the strong color of the AuNPs, the original transparency of the dressings was lost, complicating noninvasive ocular inspection of the wounds. To investigate possibilities to circumvent this issue, we explored strategies to formulate the AuNP-functionalized BC fibrils into a printable ink, allowing us to pattern the BC-stabilized AuNPs within CNF dressings using a 3D printing approach (Figure 2e,f). To facilitate ink formulation, the BC membranes were dissociated by sonication before the addition of AuNPs. The dissociated BC retained its nanofibrillar structure, and the adsorption of AuNPs (ø 50 nm) resulted in fibrils decorated with AuNPs, forming red-colored suspensions with a well-defined LSPR band (Figure 2g,h). The CNF dressings exhibit similar physicochemical characteristics as BC,^50,51^ and by printing the BC-AuNP suspension in the CNF dressing during the templating process, we were able to retain partial transparency of the dressings while enabling AuNP-based sensing.

**Figure 2:**
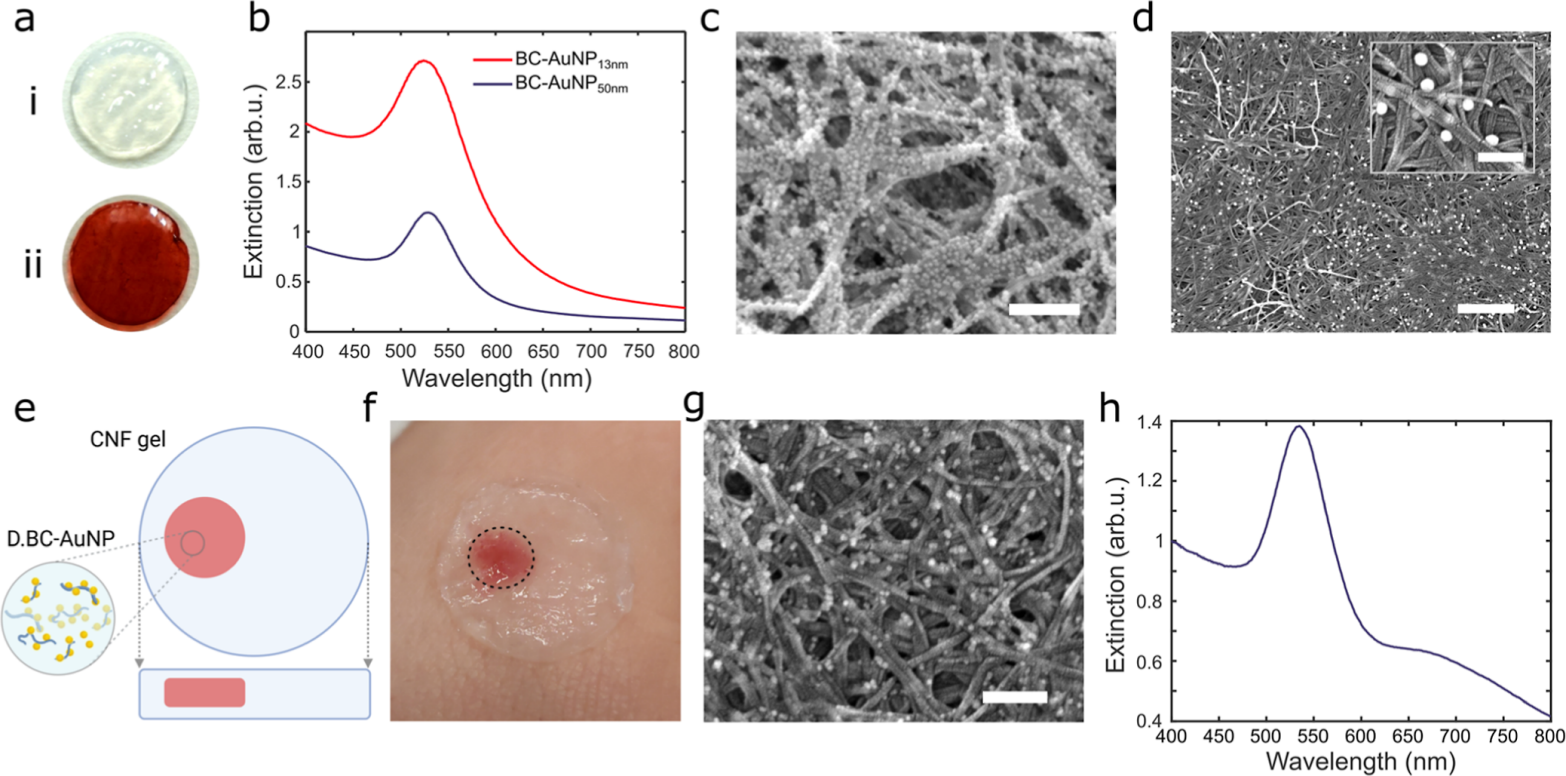
(a) (i) Photograph of pristine BC. (ii) Photograph of composites where AuNPs have self-assembled to BC to form BC-AuNPs. (b) UV–vis spectra of BC-AuNP_13 nm_ and BC-AuNP_50 nm_. (c) SEM micrographs of BC-AuNP_13 nm_. Scale bar: 500 nm (d) SEM micrographs of BC-AuNP_50 nm_. Scale bars: 1 μm, scale bar inset: 200 nm. (e) Spatial patterning of sensors was accomplished by 3D printing of a BC-AuNP (D.BC-AuNP) ink in wood-derived CNF-based wound dressings. The large square/circle indicates the side/top view of the dressing, and the small square/circle indicates the positioning of the printed D.BC-AuNP ink. (f) Photograph of the 3D-printed sensor integrated in CNF hydrogel dressings applied to skin in ambient light. (g) SEM micrograph of D.BC-AuNP 50 nm. Scale bar: 200 nm. (h) UV–vis spectra of D.BC-AuNP 50 nm.

### Functionalization of BC-AuNPs with Cas-Cy3

To enable monitoring of protease activity, the AuNPs adsorbed on the BC fibrils were further functionalized with a protease substrate labeled with cyanine3 (Cy3) (Figure 3a). By positioning the Cy3 dye sufficiently close to the AuNP surface, the fluorophore will be quenched as a result of nonradiative energy transfer processes, including Förster resonance energy transfer (fluorescence resonance energy transfer (FRET)) and nanoparticle surface energy dissipation.^58,59^ Proteolytic cleavage of the protease substrate can then release the fluorophore, resulting in an increase in the fluorescence intensity.^60^ We first explored the possibility of using bovine casein (Cas) as a protease substrate. Cas contains multiple recognition sites for relevant proteases and is widely used as a generic substrate for detection of total protease activity.^61,62^ Cas was immobilized on the AuNPs by physisorption followed by Cy3 labeling using Cy3-*N*-hydroxysuccinimide (NHS). The adsorption of Cas on the AuNPs resulted in a distinct red-shift of the LSPR band (Δ*λ* = 3.3–4.5 nm) for both the 13 and 50 nm AuNPs due to the change in refractive index in the vicinity of the AuNP surface, indicating formation of a protein monolayer on the surface of the nanoparticles (Figure 3b,c). An additional red-shift of approximately 1–2 nm of the LSPR band was observed after Cy3 conjugation (Figure 3b,c), likely due to both an additional change in refractive index and an overlap of the LSPR band with the absorption band of the dye. The conjugation of Cy3 to the immobilized Cas resulted in efficient radiative quenching because of the small separation between the dyes and the AuNP surface (Figure 3d,e). The dressings were carefully rinsed to remove any unbound dye and then exposed to trypsin as a model protease. After 60 min incubation with 1 mg/mL trypsin, the proteolytic cleavage of the immobilized Cas-Cy3 resulted in a 1.4–4.9-fold increase in fluorescence intensity of the dressings (Figure 3e). The largest increase was seen for BC-AuNP_13 nm_-Cas-Cy3, which is most likely a result of the higher surface concentration of the 13 nm AuNPs and consequently higher concentrations of Cas-Cy3 in the dressings. The possibility of detecting other proteases was confirmed using collagenase type I (Col-1) (Figure 3f). The fluorescence increase was slightly smaller for Col-1 compared to trypsin, indicating lower proteolytic activity of Col-1 when using Cas as a substrate.

**Figure 3:**
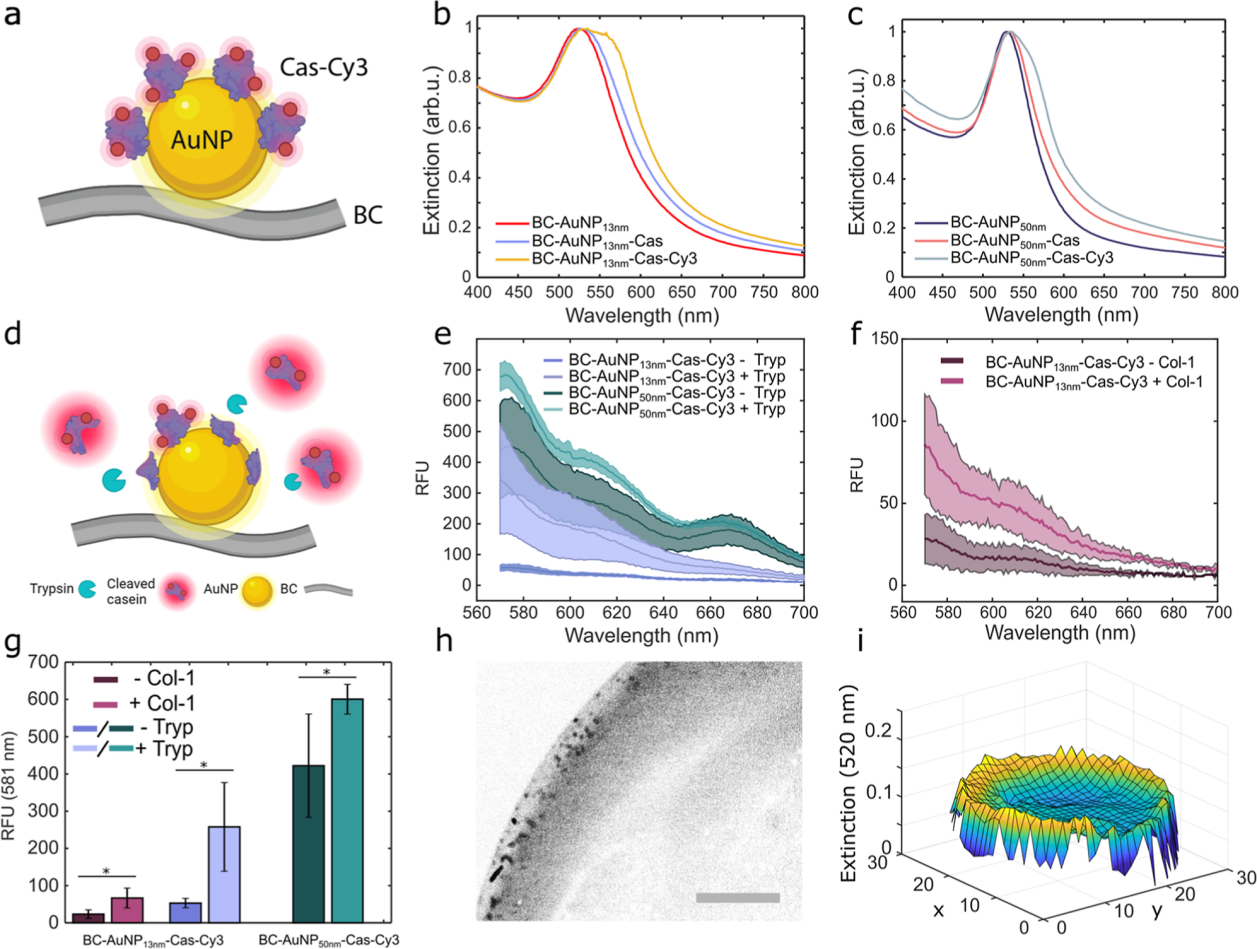
(a) Schematic illustration of the functionalization of BC-AuNP with a Cy3-labeled protease substrate. (b) UV–vis spectra of BC-AuNP_13 nm_ before and after immobilization of Cas and conjugation of Cy3. (c) UV–vis spectra of BC-AuNP_50 nm_ before and after immobilization of Cas and conjugation of Cy3. (d) Schematic illustration showing the sensor principle: Cas-Cy3 fluorescence quenching and dequenching due to cleavage of Cy3-functionalized Cas by the model protease trypsin. Fluorescence spectra of BC-AuNP_13/50 nm_-Cas-Cy3 before and after incubation with (e) trypsin (1 mg/mL, 60 min at 37 °C) and (f) collagenase type 1 (0.5 mg/mL, 60 min at 37 °C), and (g) corresponding fluorescence intensities at 581 nm. (h) Background subtracted fluorescence microscopy images converted to binary (black and white) showing the increase in fluorescence (black pixels) of BC-AuNP_50 nm_-Cas-Cy3 after incubation with trypsin (1 mg/mL, 60 min at 37 °C). Scalebar: 0.5 mm. (i) Extinction mapping of a BC-AuNP membrane at 520 nm. Shaded areas in (e,f) and error bars in (g) show standard deviations. *n* ≥ 3, *p < 0.05.

Although Cas is often used as a generic protease substrate for detection of protease activity, it is a fairly large protein with a molecular weight of 25–30 kDa.^63^ Since quenching through resonance energy transfer is highly distance dependent,^60^ some of the fluorophores could be positioned outside the range for efficient quenching when using Cas as a substrate. Cas can also form larger micelles of varying sizes, depending on concentration and buffer conditions, such as ionic strength and pH.^64^ Since both Cas monomers and micelles of different sizes are likely adsorbed onto the AuNPs, the distance between the conjugated Cy3 and the AuNPs will not be uniform. Cas-Cy3 adsorbed to native BC showed a weak fluorescence signal that did not change when exposed to trypsin, which supports the use of AuNPs for efficient quenching (Figure S1, Supporting Information). A certain degree of background fluorescence was thus to be expected to be caused by fluorophores that were located too far from the AuNP surface to be quenched.

The background fluorescence was more pronounced for BC functionalized with AuNP_50 nm_ (BC–AuNP_50 nm_-Cas-Cy3) compared to BC with AuNP_13 nm_ (BC–AuNP_13 nm_-Cas-Cy3) (Figure 3g). This finding indicates that Cas either obtained different conformations when absorbed to AuNPs of different sizes or that the higher surface density of AuNP_13 nm_ on the BC fibrils contributed to improved quenching since a higher surface density of AuNPs could increase the probability of having a nanoparticle in the vicinity of a fluorophore, resulting in more efficient quenching of Cas-Cy3. Moreover, since BC is a biological material produced in a biotechnical process, spatial variations in the thickness of the BC dressings can influence the number AuNPs adsorbed, which could contribute to variations in the background signal. Despite some background fluorescence, fluorescence imaging of the BC-AuNP_50 nm_-Cas-Cy3 dressings showed a distinct increase in the fluorescence after exposure to trypsin (Figures 3h, and S2, Supporting Information). Pixel analysis revealed a clear shift in red pixel value after addition of trypsin, while the number of red pixels remained constant, indicating homogeneous cleavage of Cas-Cy3 and an overall increase in fluorescence. The fluorescence increase was more pronounced at the rim of the BC dressings after exposure to proteases (Figure 3h), which likely is due to the higher concentration of AuNPs at the dressing perimeter due to diffusion occurring from multiple directions during the assembly and functionalization processes. This was corroborated by mapping the AuNP extinction during the early stages of the self-assembly process, where the extinction at 520 nm was found to be markedly higher around the rim (Figure 3i). Likewise, diffusion of trypsin into the dressings is expected to be higher at the rim compared to when diffusion only can occur from the top or bottom of the dressings.

### Protease Detection Using Peptide-Based Substrates

To further optimize the performance of the protease-responsive dressings and reduce the background fluorescence, we explored the possibilities of using a significantly smaller (*M*_w_ = 2.2 kDa) polypeptide-based protease substrate (CPI2, Figure 4a) as an alternative to Cas. CPI2 is designed to be cleaved by several matrix metalloproteinases (MMPs) and bacterial proteases,^65^ making it highly relevant for assessing the protease activity in wounds. To first evaluate the performance of CPI2 as a protease substrate and confirm its cleavage by the model protease Col-1, we used a simplified model system based on FRET to avoid additional complications of the BC-AuNP dressings. To do this, CPI2 was first synthesized with the FRET pair Cy3 and Cy5 at the N- and C-termini, respectively (Figure 4a). The resulting Cy3-CPI2-Cy5 peptide showed distinct Cy5 emission at 671 nm when excited at the Cy3 absorption band (535 nm), confirming FRET (Figure 4b). When exposed to Col-1, cleavage of Cy3-CPI2-Cy5 resulted in a significant decrease in Cy5 emission, while the Cy3 emission was restored, clearly demonstrating that the peptide was cleaved by Col-1 (Figure 4b,c). For integration in the wound dressings, CPI2 was synthesized with a cysteine (Cys) residue at the C-terminus followed by a short β-alanine spacer to avoid steric hindrance that could impair proteolytic cleavage. A Cy3 fluorophore was included at the N-terminus during peptide synthesis, resulting in the peptide Cy3-CPI2-C (Figure 4a).^66^ The Cys residue enabled directional and site-specific immobilization of the Cy3-CPI2-C peptide on AuNPs, resulting in a maximum theoretical distance of the Cy3 fluorophore of about 6.5 nm from the AuNP surface when the polypeptide is fully extended (calculated as a rigid rod). However, most likely, the average distance is shorter than this due to the conformational flexibility of the peptide, and the fluorophore should hence be optimally positioned for efficient quenching. Accordingly, very limited fluorescence was observed when Cy3-CPI2-C was immobilized on BC-AuNP_50 nm_. Moreover, no increase in fluorescence was seen after addition of trypsin to BC-AuNP_50 nm_-CPI2-Cy3 (Figure 4d,e), which was expected, as trypsin cleaves peptide bonds at the C-terminal side of lysine (Lys) and arginine (Arg) residues unless preceded by a proline (Pro) residue. Cy3-CPI2-C does not contain any Lys or Arg residues and should thus not be cleaved by trypsin. However, a significant fluorescence intensity increase was observed upon the addition of Col-1 (Figure 4e), indicating cleavage and release of the Cy3. The fluorescence recovery was relatively quick (<30 min), indicating little or no diffusion limitation and low steric hindrance at the AuNP surface. The fluorescence intensity increased slightly with longer incubation times (Figure 4f).

**Figure 4:**
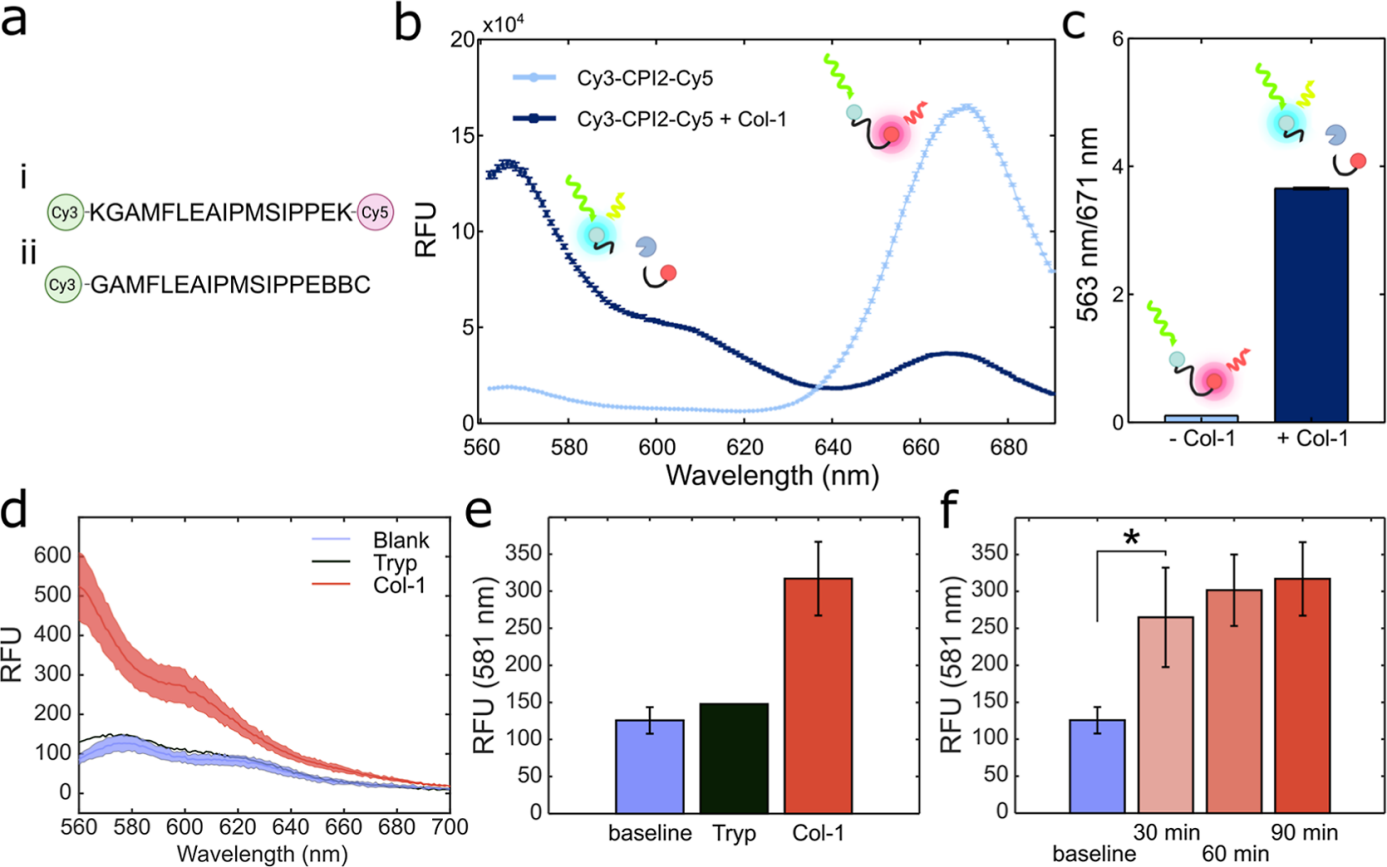
(a) Amino acid sequence of (i) FRET protease substrate peptide Cy3-CPI2-Cy5 and (ii) protease substrate peptide Cy3-CPI2-C. (b) Fluorescence emission spectra and (c) ratio of emissions at 563 and 671 nm of Cy3-CPI2-Cy5 before and after the addition of Col-1 (1 mg/mL). (d) Fluorescence spectra and (e) emission at 581 nm of BC-AuNP_50 nm_-CPI2-Cy3 before and after exposure to Col-1 or trypsin after 90 min of incubation at RT and (f) before and after exposure to Col-1 over a period of 90 min. Shaded areas and error bars show standard deviations, *n* ≥ 3, *p < 0.05.

### Sensor Response to Wound Pathogens

To assess the sensor response to bacterial proteases from relevant wound pathogens, the dressings were incubated with supernatants from cultures of S. aureus and P. aeruginosa, which are among the most common pathogens isolated from chronic wounds.^67^ Both bacteria are known to produce virulence factors, including proteases,^42^ that impair wound healing, and possess intrinsic and acquired antibiotic resistance, complicating clinical infection management.^68^ When BC-AuNP_50 nm‑_CPI2-Cy3 dressings were exposed to supernatants from cultures of S. aureus or P. aeruginosa in the stationary phase (10^8^–10^9^ CFU/mL), a significant increase in fluorescence intensity of the dressings were observed compared to the negative control (Lysogeny broth (LB) medium), Figure 5a. In wounds, bacterial colonization typically exceeds 10^4^–10^5^ CFU/mL, often leading to infection and impaired healing.^69,70^ A more rapid fluorescence increase was seen for dressings exposed to S. aureus supernatants compared to P. aeruginosa, indicating higher proteolytic activity in the former (Figure 5b). When incubating the dressings in samples containing the growing bacteria, no fluorescence increase was seen during the first 9 h for both bacteria, corresponding to the lag and early exponential phase, indicating low proteolytic activity (Figure 5c,d). After about 9–10 h, the fluorescence intensity started to increase, which coincided with the late exponential and postexponential phases of growth of the bacteria. For both S. aureus and P. aeruginosa, protease production and activity are closely linked to the bacterial growth phase.^71,72^ In S. aureus, the synthesis of extracellular proteases is activated by the accessory gene regulator (agr) system during the late exponential and postexponential phases of growth.^67,71^ In P. aeruginosa, protease activity is upregulated in the late exponential to stationary phases, driven by quorum-sensing systems that respond to cell density and environmental cues.^73^ This regulation ensures that proteolytic enzymes are produced when bacterial populations are high and resources may be limited, enhancing the pathogens’ ability to damage host tissues and evade immune responses. The observed fluorescence increase thus aligns with the known regulation of bacterial protease expression, which is upregulated in the late exponential and stationary phases. Moreover, the absence of a fluorescence increase during the lag and early exponential phases, despite active bacterial growth, suggests that the sensor response is not directly proportional to bacterial concentration but rather reflects the bacterial growth stage and the onset of virulence-associated activity. S. aureus secretes several extracellular proteases, including the metalloproteinase aureolysin, the serine glutamyl endopeptidase SspA, and two related cysteine proteinases (staphopain, ScpA and SspB).^43^ Proteases secreted by P. aeruginosa include elastase A (LasA), elastase B (LasB), alkaline protease, protease IV (PIV), Pseudomonas small protease (PASP), large protease A (LepA), MucD, and P. aeruginosa aminopeptidase (PAAP).^45^ In addition, the gram-negative P. aeruginosa has over 40 proteases and peptidases located in the cell envelope,^44^ including the periplasmic space and the outer lipid membrane. Since the sensor detects overall protease activity, it is not capable of discriminating specific bacteria. While the casein substrate detects general proteolytic activity, CPI2 was designed to be cleaved specifically by bacterial proteases and MMPs, reflecting the increased protease activity characteristic of infected wounds rather than normal wound healing. As a simple and noninvasive wound dressing-integrated sensor for early detection of infection, this strategy can enable better possibilities to rapidly assess wound status and optimize treatment.

**Figure 5:**
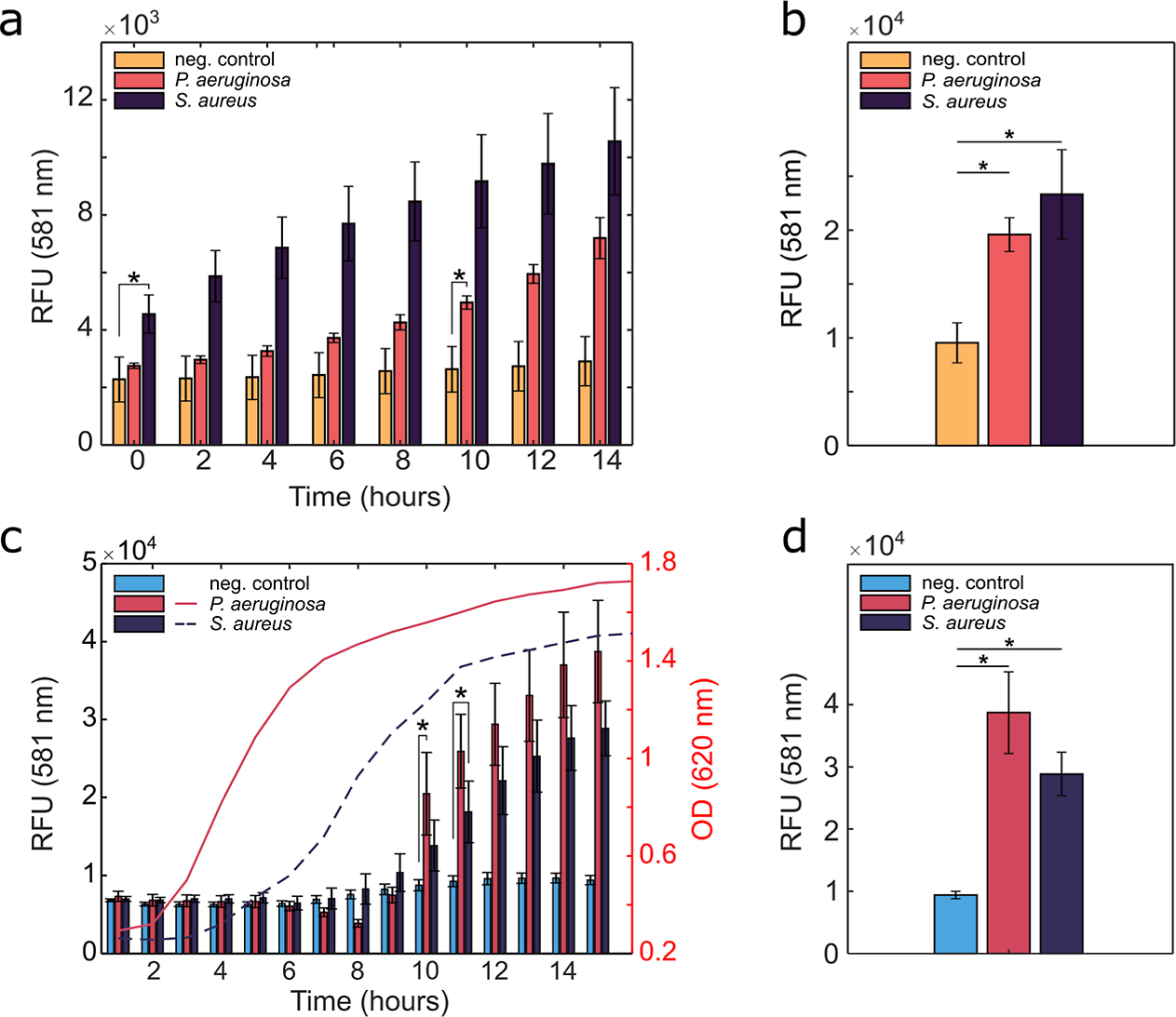
Fluorescence recovery over (a) 0–14 h and after (b) 20 h for BC-AuNP_50 nm_-CPI2-Cy3 incubated with LB medium (negative control) or supernatants of S. aureus and P. aeruginosa cultures. Fluorescence recovery over (c) 6–15 h and after (d) 20 h for BC-AuNP_50 nm_-CPI2-Cy3 incubated in LB medium (negative control) or in suspensions of cultured S. aureus and P. aeruginosa. All data show fluorescence intensity at 581 nm. Error bars show standard deviations. *n* = 5 for S. aureus and P. aeruginosa and *n* = 4 for negative control in (a,b) and *n* ≥ 2 for (c,d), *p < 0.05.

## Conclusions

In summary, a strategy for integrating fluorescence turn-on sensors for detection of protease activity in nanocellulose wound dressings has been developed. BC was functionalized with AuNPs by exploiting the strong van der Waals interaction between the nanocellulose fibrils and nanoparticles when suppressing electrostatic repulsion at slightly elevated ionic strength. The BC-AuNPs could be integrated either homogeneously in the dressing or by patterning in CNF wound dressings using 3D printing to enable protease sensing while retaining the transparency offered by the nanocellulose dressings allowing for ocular wound inspection. The AuNPs were further modified with fluorescence-labeled protease substrates. Two different substrates were investigated: Cas-Cy3 and the polypeptide Cy3-CPI2-C. Quenching and fluorescence turn-on upon exposure to trypsin or Col-1 were seen for both substrates. The background fluorescence was, however, larger in the case of Cas-Cy3, probably due to the larger size of the protein and thus larger average separation between Cy3 and the AuNP surface compared to the case of Cy3-CPI2-C. When subjecting the protease-responsive dressings to supernatants from cultures of S. aureus and P. aeruginosa, significant fluorescence recovery was seen for both bacteria. A drastic increase in fluorescence intensity of the dressings was also seen when exposing the dressings to bacteria suspensions, resulting in a time-dependent fluorescence turn-on signal with the possibility to detect protease activity for growing bacteria in real-time. An increase in proteolytic activity was seen during the late exponential to stationary phases of bacteria growth, which is when protease production is typically upregulated for both S. aureus and P. aeruginosa. Because protease production by wound pathogens is tightly regulated by environmental and cell-density-dependent factors, the sensor response reflects the onset of virulence-associated activity rather than a direct linear function of bacterial concentration. While this study focuses on in vitro validation, future work will include testing in simulated wound environments, such as artificial wound exudates or ex vivo wound models, to further assess the performance of the sensors under physiologically relevant conditions. The fabrication approach is compatible with scalable manufacturing as BC is already used in commercially available wound dressings and the incorporation of gold nanoparticles through self-assembly or 3D printing allows for cost-effective and flexible sensor integration. The proposed strategy allows for rapid readout of protease activity using wound dressing-integrated sensors, which can allow for assessment of wound status and guide healthcare personnel on the need for interventions without the unnecessary disruption of the healing process caused by removal of the dressings.

## Materials and Methods

### General

All chemicals were purchased from Merck KGaA and used without further purification unless otherwise noted. AuNPs (ø 50 nm) were purchased from BBI Solutions (Crumlin, UK).

### Synthesis of AuNPs

AuNPs with an average diameter of ca. 13 nm were synthesized by citrate reduction of HAuCl_4_ (Turkevich-method).^74^ All glassware were cleaned by heating a solution of 25% NH_3_, 30%H_2_O_2_ and Milli-Q Water (18.2 MΩ cm^–1^) (Milli-Q), mixed 1:1:5, to 85 °C for 15 min. The glassware was then thoroughly rinsed in Milli-Q. A 50 mL solution of 1 mM HAuCl_4_ was brought to a rolling boil. The reduction reaction was initiated by rapidly adding 20 mL of 38.8 mM citrate solution while stirring vigorously. The mixture was left to reflux for approximately 15 min and cooled to room temperature.

### Self-Assembly of AuNPs in BC

BC produced by Komagataeibacter xylinus was obtained from S2Medical AB (Linköping, Sweden) and was cut into circular membranes using a 6 mm biopsy punch prior to functionalization. BC-AuNP_13 nm_ was prepared by immersing BC in a suspension of AuNPs obtained by mixing 500 μL of AuNPs (13 nm,13 nM) with 500 μL of citrate buffer 10 mM, pH 6. BC-AuNP_50 nm_ was prepared by immersing BC in 1 mL stock suspension of AuNPs (50 nm) with a concentration of 7.5 × 10^–14^ M. Incubation for both particle types was carried out for 5 days on an orbital shaker. After 5 days of incubation, the dressings were rinsed and stored in Milli-Q until further use. UV–vis spectra were obtained using a microplate reader (Tecan Infinite M1000 Pro, Tecan Austria GmbH, Grödig/Salzburg, Austria).

### Dissociation of BC through Sonication

D.BC was prepared by placing BC membranes in a glass vial together with 1 mL of Milli-Q per ø 6 mm BC membrane and sonicated for 15 min using a probe tip sonicator (Bandelin Electronic GmbH & CO. KG, Berlin, Germany).

### CNF Printing and Patterning

A CNF suspension of 0.25 wt % was prepared from TEMPO-oxidized softwood nanofibrils, as described in a study by Baş, et al.^51^ To homogenize the CNF suspension, 1.5 mL was extruded between two syringes connected by a Luer lock for 20 cycles. The homogenized CNF suspension was added to a filter tube prepped with a PVDF Durapore filter featuring 0.2 μm pores (3 M, Maplewood, MN, US) and vacuum filtrated until a dry membrane had formed. On top of the membrane, 1 mL of 0.25 wt % of additional homogenized CNF suspension was added. D.BC-AuNP was prepared by mixing dissociated BC with 1 mL of AuNP (ø 50 nm) suspension and vortexing the mixture for 1 min. The D.BC-AuNP was subsequently mixed with 100 μL of 0.77 wt % CNF suspension to form an ink. The ink was printed into the wet CNFs on top of the BC membrane using a syringe with a 1.2 mm diameter needle.

### Extinction Mapping of BC-AuNPs

BC-AuNP membranes were prepared by immersing BC membranes (ø 6 mm) in 300 μL of 2.6 nM AuNP (ø 13 nm) suspension for 5 days. BC-AuNP extinction at 520 nm was probed using a plate reader (CLARIOstar, BMG Labtech, Ortenberg, Germany) in a 30 × 31 matrix.

### Peptide Synthesis

The peptide Cy3-CPI2-C with the sequence H_2_N-GAMFLEAIPMSIPPEBBC–CONH_2_ where B is β-Ala and the FRET peptide Cy3-CPI2-Cy5 with the sequence Ac–K­(Mtt)­GAMFLEAIPMSIPPEK­(Alloc)-CONH_2_ were synthesized on an automated peptide synthesizer (Liberty Blue, CEM, Matthews, US). ProTide rink amide resin (0.19 mmol/g) was used as a solid support, and the peptides were synthesized in a 0.05 μmol scale using standard Fmoc chemistry. Amino acids were sequentially coupled to the growing peptide using a 5-fold excess of Fmoc-protected amino acid, *N*,*N*′-diisopropylcarbodiimide (DIC) and a 10-fold excess of Oxyma Pure as a base. The reaction mixture was heated to 90 °C using microwaves and allowed to proceed for 2 min. Sequential Fmoc deprotection was performed using 20% piperidine in DMF (v/v) at 90 °C for 1 min. After attachment of all amino acids and final Fmoc deprotection, 0.01 μmol H_2_N-GAMFLEAIPMSIPPEBBC–CONH_2_ resin was reacted with Cyanine3 *N*-hydroxy succinimide ester (Cy3-NHS) dye (0.01 μmol, Lumiprobe GmbH, Hannover, Germany) in DMF for 3 h. The N-terminal of H_2_N–K­(Mtt)­GAMFLEAIPMSIPPEK­(Alloc)-CONH_2_ was acetylated using acetic anhydride/DMF (1:1, v/v, 20 mL) for 1 h before 0.005 μmol resin was labeled with the FRET pair Cy3 and Cy5. Therefore, Alloc deprotection was performed using a molar ratio of peptide/Pd­(PPh_3_)_4_/PPh_3_:*N*-methylaniline (1:0.5:5:10) in dry THF that was incubated overnight. Afterward the resin was washed with 0.5% *N*,*N*-diisopropylethylamine (DIPEA) in DMF, 0.5% sodium diethyldithiocarbamate in DMF, followed by pure DMF and DCM. A sulfo-Cy3-NHS-esther (0.01 μmol, Lumiprobe GmbH, Hannover, Germany) was then coupled to the deprotected lysine residue in DMF containing 0.05 μmol DIPEA. Mtt deprotection of the second lysine residue was performed by a stepwise addition of a 1% TFA in DCM solution followed by washing with 1% DIPEA in DCM. For the coupling of sulfo-Cy5-NHS ester (0.01 μmol, Lumiprobe GmbH, Hannover, Germany), the same process as for Cy3 was used. The crude peptides were cleaved from their solid supports by treatment with trifluoracetic acid (TFA): triisopropylsilane: Milli-Q (95:2.5:2.5, v/v/v) for 3 h. The cleavage cocktail was concentrated under a stream of nitrogen, and the crude peptide was precipitated twice in cold diethyl ether. Purification was performed on a reversed phase column (C18, 120 Å, 5 μm, ReproSil Gold) attached to a semipreparative high-performance liquid chromatography (HPLC) system (Ultimate 3000, Dionex, Sunnyvale, USA) using an aqueous gradient of acetonitrile and 0.1% TFA as buffer. Peptide identity was confirmed using matrix-assisted laser desorption/ionization time-of-flight mass spectrometry (MALDI-TOF MS, ultrafleXtreme, Bruker, Billerica, MA, USA) running in a positive ionization mode with alpha-cyano-4-hydroxycinnamic acid as a matrix (Figure S3a,b, Supporting Information). Peptide purity was confirmed using the same HPLC-system and column, as described above (Figure S3c,d and Supporting Information).

### FRET-Assay

Cy3-CPI2-Cy5 (14 μM, 100 μL of PBS) was mixed with 100 μL of Col-1 (1 mg/mL) in PBS or 100 μL of PBS as a negative control. After 1.5 h incubation at 37 °C the fluorescence spectra were recorded using a microplate reader (CLARIOstar, BMG Labtech, Germany), where samples were excited at 535 nm and emission was measured at 550–960 nm. The peptide concentration was calculated according to the Beer–Lambert law through absorption of Cy3 at 548 nm and Cy5 at 646 nm.

### Functionalization of BC-AuNPs

Casein (Cas) was dissolved in Milli-Q water through continuous adjustment of pH to pH > 10 with NaOH by gentle and repeated heating and careful shaking. BC-AuNPs were functionalized incubated in 200 μL of 1 mg/mL Cas solution overnight on an orbital shaker, thoroughly rinsed with Milli-Q water on an orbital shaker for 1 h. Sulfo-Cyanine3 NHS ester (Cy3-NHS) was dissolved in DMSO and diluted to 5 μM in Milli-Q water. BC-AuNP-Cas were incubated with 100 μL of Cy3-NHS and incubated for 1 h. The resulting BC-AuNP-Cas-Cy3 was washed in a large volume (approximately 10 mL per 6 mm dressing) of PB 10 mM pH 7.4 for 1 h on an orbital shaker. Samples were stored in PB 10 mM, pH 7.4. For functionalization of BC-AuNPs with Cy3-CPI2-C, the peptide was diluted to 5 μM in PBS to a final volume of 200 μL, added to BC-AuNPs in Eppendorf tubes, and incubated overnight on an orbital shaker, followed by rinsing in 1 mL of PBS 10 mM pH 7.4 for 15 min on an orbital shaker, after which the buffer was exchanged for new PBS and incubated for another 15 min. The dressings were protected from light during and after functionalization with Cas-Cy3 and Cy3-CPI2-C to avoid bleaching.

### Protease Sensing

The protease-responsive dressings were exposed to a solution of trypsin or collagenase type I (200 μL, 0.5–1 mg/mL in PBS 10 mM, pH 7.4) and incubated for 1 h. Extinction and fluorescence spectra were obtained before and after the addition of protease.

### Detection of Protease Activity in Bacteria Supernatants

Bacterial supernatants were obtained by culturing S. aureus (ATCC 29213, MSSA, ATCC, Manassas, VA) and P. aeruginosa, received from the Department of Laboratory Medicine at Örebro University Hospital, overnight in LB at 37 °C. Approximately 1.5 mL of bacterial culture was added to an Eppendorf tube and centrifuged at 3000 rpm for 3 min. The supernatant (200 μL) was added to AuNP-BC-CPI2-Cy3 in 96-well microplates.

Detection of protease activity in bacteria cultures: S. aureus and P. aeruginosa were streaked on LB agar plates and incubated at 37 °C overnight. Single colonies were inoculated into 5 mL of LB broth and incubated on a shaker (400 rpm) at 37 °C overnight prior to the experiment. The bacterial concentrations were determined by viable count and were adjusted to correlate with approximately 10^9^ CFU/ml. Prior to the addition of the bacterial sample, the fluorescence spectra of the BC-AuNP_13 nm_-CPI2-Cy3 dressings were recorded. The dressings were then placed at the bottom of the wells in a 96-well plate and covered with 250 μL of bacterial suspension in LB containing approximately 10^5^ CFU/mL. The fluorescence (581 nm) was then recorded at 0, 30, 60, 90, and 120 min using a microplate reader (BioTek Synergy H1, Agilent, Santa Clara, CA, USA). Between measurements, the plate was placed on a shaker, protected from light. After the first 120 min, the fluorescence was recorded every 2 h for the remainder of the experiment, with orbital shaking for 30 s between measurements. The temperature in the reader was set to 37 °C.

## Supplementary Material



## Notes

Additional fluorescence spectra of dressings, fluorescence microscopic images of dressings before and after addition of proteases, and HPLC and MALDI-ToF data on peptide identity and purity (PDF)
