# Supplementary material for: Nanocellulose Wound Dressings with Integrated Protease Sensors for Detection of Wound Pathogens
Source: ACS Sens. 2025 May 20;10(6):3953–63. doi: 10.1021/acssensors.4c03428 (PMC12210249; doi:10.1021/acssensors.4c03428)
Supplement: Supplementary file 1 [file se4c03428_si_001.pdf]

Supporting Information

Nanocellulose Wound Dressings with Integrated Protease Sensors for Detection of Wound Pathogens

Olof Eskilson[1], Emanuel Wiman[2], Nina Reustle[1], Jakob Langwagen[1], Zeljana Sotra[3], Anna Svärd[2], Robert Selegård[1], Yağmur Baş[4], Linn Berglund[4], Kristiina Oksman[4], Torbjörn Bengtsson[2], Johan P. E. Junker[3,5], Hazem Khalaf[2], and Daniel Aili[1,]*

[1] *Laboratory of Molecular Materials, Division of Biophysics and Bioengineering, Department of Physics, Chemistry and Biology (IFM), Linköping University, Linköping SE-58183, Sweden.*

[2] *Unit of Microbiology, Immunology and Reproductive Science, School of Medical Sciences, Faculty of Medicine and Health, Örebro University, 703 62 Örebro, Sweden.*

[3] *Centre for Disaster Medicine and Traumatology, Department of Biomedical and Clinical Sciences, Linköping University, 581 85 Linköping, Sweden.*

[4] *Division of Materials Science, Department of Engineering Sciences and Mathematics, Luleå University of Technology, 971 87 Luleå, Sweden.*

[5] *Laboratory for Experimental Plastic Surgery, Department of Biomedical and Clinical Sciences, Linköping University, 581 85 Linköping, Sweden.*

* Corresponding author: daniel.aili@liu.se

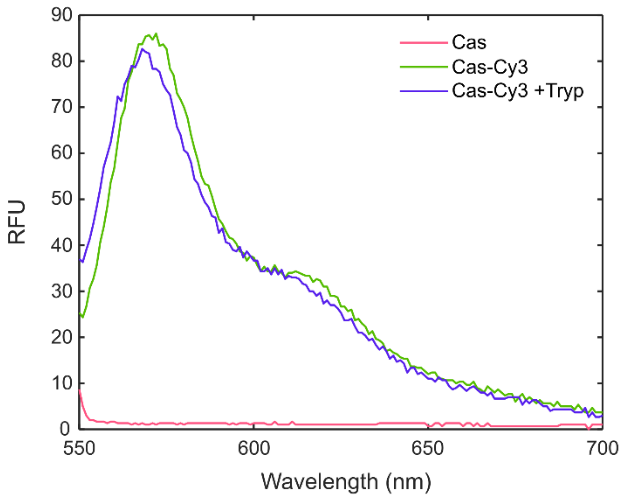

Figure S1. Fluorescence intensity of BC-Cas, and BC-Cas-Cy3 before and after incubation in 100 µL 0.5 mg/mL trypsin in PBS for 30 min.

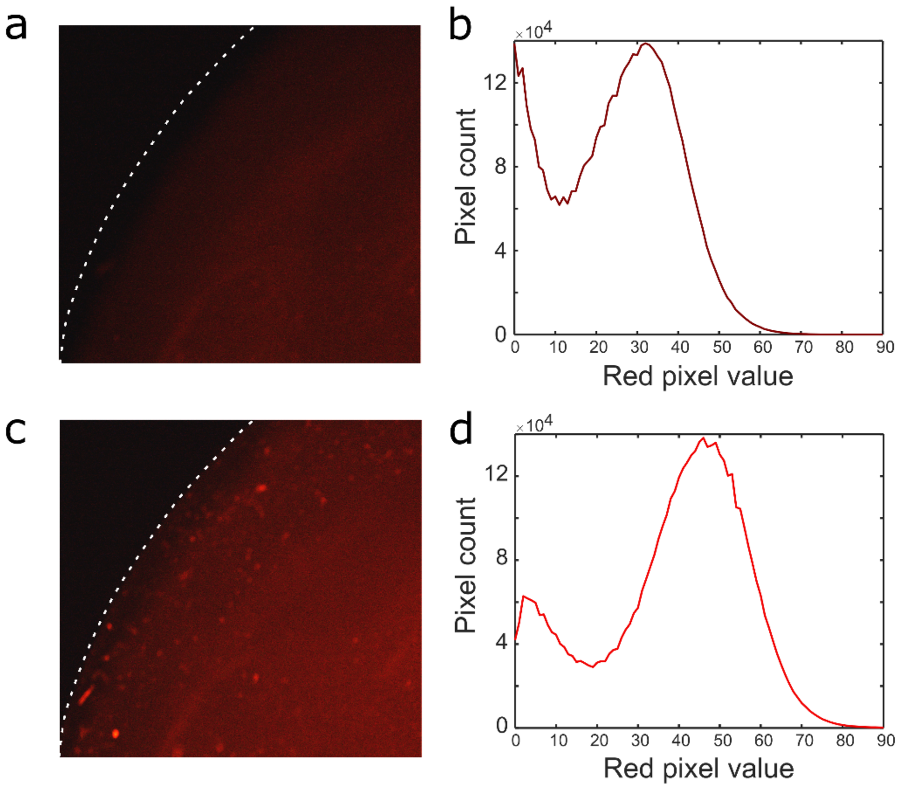

Figure S2. a,c) Fluorescence microscopy of BC-AuNP$_{50nm}$-Cas-Cy3 and b,d) pixel count analysis, (a,b) before, and (c,d) after addition of trypsin (1 mg/mL, incubated for 1 h at 37 °C).

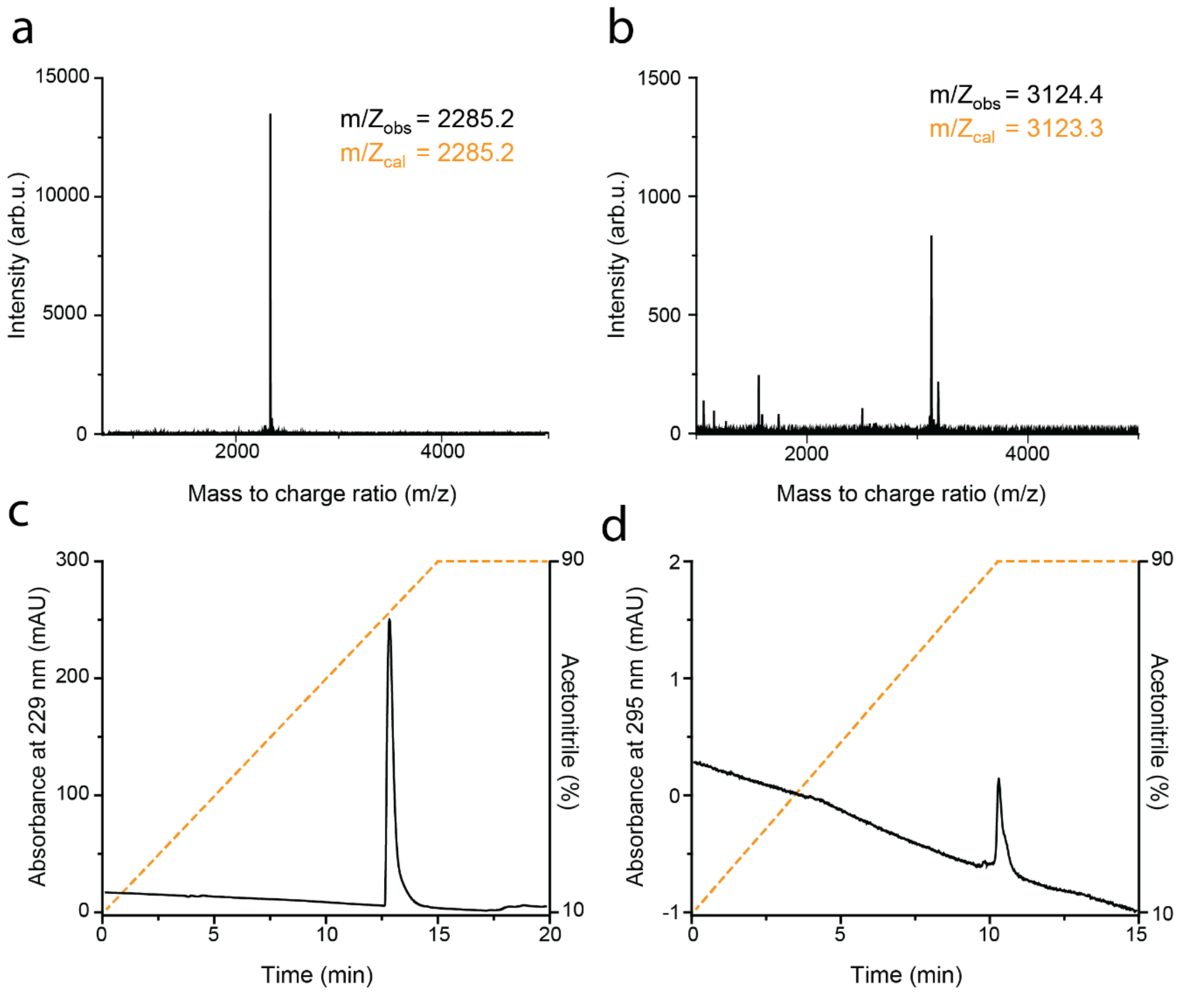

Figure S3. Peptide identity and purity. Peptide mass of a) Cy3-CPI2-C and b) Cy3-CPI2-Cy5 acquired using MALDI-TOF running in positive ionization mode. Chromatogram of c) Cy3-CPI2-C and d) Cy3-CPI2-Cy5 with orange dotted line indicating the acetonitrile gradient used.
